# The Impact of the Extracellular Matrix Environment on *Sost* Expression by the MLO-Y4 Osteocyte Cell Line

**DOI:** 10.3390/bioengineering9010035

**Published:** 2022-01-13

**Authors:** Robert T. Brady, Fergal J. O’Brien, David A. Hoey

**Affiliations:** 1 Tissue Engineering Research Group, Department of Anatomy & Regenerative Medicine, Royal College of Surgeons in Ireland, D02 YN77 Dublin, Ireland; robertthomasbrady@gmail.com (R.T.B.); fjobrien@rcsi.ie (F.J.O.); 2 Trinity Centre for Biomedical Engineering, School of Engineering, Trinity College Dublin, D02 PN40 Dublin, Ireland; 3 Advanced Materials and BioEngineering Research Centre (AMBER), Trinity College Dublin & Royal College of Surgeons in Ireland, D02 PN40 Dublin, Ireland; 4 Department of Mechanical, Manufacturing, and Biomedical Engineering, Trinity College Dublin, D02 PN40 Dublin, Ireland

**Keywords:** osteocyte, MLO-Y4, bone, composition, dimensionality, 3D, fluid flow, mechanobiology

## Abstract

Bone is a dynamic organ that can adapt its structure to meet the demands of its biochemical and biophysical environment. Osteocytes form a sensory network throughout the tissue and orchestrate tissue adaptation via the release of soluble factors such as a sclerostin. Osteocyte physiology has traditionally been challenging to investigate due to the uniquely mineralized extracellular matrix (ECM) of bone leading to the development of osteocyte cell lines. Importantly, the most widely researched and utilized osteocyte cell line: the MLO-Y4, is limited by its inability to express sclerostin (*Sost* gene) in typical in-vitro culture. We theorised that culture in an environment closer to the in vivo osteocyte environment could impact on *Sost* expression. Therefore, this study investigated the role of composition and dimensionality in directing *Sost* expression in MLO-Y4 cells using collagen-based ECM analogues. A significant outcome of this study is that MLO-Y4 cells, when cultured on a hydroxyapatite (HA)-containing two-dimensional (2D) film analogue, expressed *Sost*. Moreover, three-dimensional (3D) culture within HA-containing collagen scaffolds significantly enhanced *Sost* expression, demonstrating the impact of ECM composition and dimensionality on MLO-Y4 behaviour. Importantly, in this bone mimetic ECM environment, *Sost* expression was found to be comparable to physiological levels. Lastly, MLO-Y4 cells cultured in these novel conditions responded accordingly to fluid flow stimulation with a decrease in expression. This study therefore presents a novel culture system for the MLO-Y4 osteocyte cell line, ensuring the expression of an important osteocyte specific gene, *Sost*, overcoming a major limitation of this model.

## 1. Introduction

Bone is a complex multicellular organ that possesses the ability to functionally adapt its structure to meet the biochemical and biophysical demands placed upon it. A potent regulator of bone adaptation is mechanical loading [1,2]. Evidence suggests that the master orchestrator of bone mechanoadaptation is the osteocyte [3,4,5], which accounts for over 90% of the cell population in mature bone [3]. These stellate cells reside within a calcified matrix, in spaces termed lacunae, and extend long processes that interconnect to coordinate secretion of biological signalling factors, such as sclerostin, regulating bone anabolism [6,7,8,9,10]. Bone extracellular matrix (ECM), formed by osteoblasts, is a composite material comprised of both organic and inorganic components. The matrix is primarily made up of collagen type I fibres that have been mineralised with inorganic hydroxyapatite (Ca_10_(PO_4_)_6_(OH)_2_) [11]. Bone ECM also comprises glycosaminoglycans (GAG) such as chondroitin sulphate and a range of growth factors [12]. The final composition of the ECM is a key factor driving the terminal differentiation of osteoblasts into osteocytes, which transition to display a completely different phenotype, gene expression, and biochemical profile. Moreover, the three dimensional ECM affords a spatial context to direct signalling events mediated by various adhesion molecules such as integrins and growth factor receptors [13]. However, this uniquely mineralised ECM also poses a fundamental obstacle in studying primary osteocytes as they are embedded in this hard mineralised tissue and, thus, are difficult to extract in sufficient numbers as they are non-proliferative ex vivo. As such, the MLO-Y4 cell line was originally developed as a model of the osteocyte and has been subsequently used extensively for this purpose with over 270 published papers having utilised this cell model [9,14]. MLO-Y4 cells share similar features with osteocytes such as dendritic processes, low alkaline phosphatase production, and high osteocalcin, and connexin 43 production [15]. The MLO-Y4 cell line has facilitated the discovery and validation of numerous mechanisms in bone, including that fluid shear stress can result in the synthesis and release of bioactive molecules that initiate signalling pathways to prevent apoptosis, recruit osteoprogenitors, and encourage the osteogenic differentiation of such cells [6,16,17,18,19].

Whilst our understanding of bone biology and the role of osteocytes have been significantly advanced through the development of the MLO-Y4 cell-line, it has several significant limitations. The primary of which, is the deficiency of sclerostin production at physiological quantities [20]. This protein is almost exclusively and constitutively produced by mature osteocytes encased in their mineralised matrix. Coded by the *Sost* gene, it acts as a negative regulator of bone formation [21] via the Wnt/β-catenin pathway [6,15]. Atkins et al. (2011) showed that sclerostin levels are markedly reduced in bones under mechanical loading and significantly increased in the unloaded bone [21,22]. Sclerostin therefore facilitates bone formation and the strengthening of existing structures at a site of loading. With our increased understanding of this molecule’s significance in regulating bone anabolism, it has become a pivotal focus of research in the development of novel anabolic therapeutics for metabolic bone disease [9,23,24]. One such therapeutic, romosozumab, is a sclerostin monoclonal antibody that was recently approved by the FDA for the treatment of osteoporosis in postmenopausal women [25]. Whilst MLO-Y4 cells remain a pivotal cell type used in investigating bone biology, the deficiency of *Sost* expression cannot be dismissed. This limitation has driven the development of the IDG-SW3 cell line, and other Sost expressing lines [9]. The IDG-SW3 cells proliferate in the presence of interferon-gamma (IFN-ɣ) under conditions of 33 °C culture [20]. Culture in 37 °C in the absence of IFN-ɣ reduces expression of T antigen that drives proliferation and the cells begin to act more like primary cells and express Sost in 2D culture after >10 days [20]. Whilst these are robust cellular models of osteocytes, the culture conditions are non-trivial and it is known that temperature changes can trigger heat shock responses and that IFN-ɣ is a potent cytokine with a diverse range of effects upon cells. Therefore, it is clear that while these models are helpful, a cell line that expresses physiological levels of sclerostin, where the regulating mechanisms are intact and without atypical growth conditions, would represent a powerful alternative.

When considering such a model for osteocyte study, it is important to consider the tenants of the tissue engineering triad. Tissue engineering has highlighted the importance of the ECM in directing the development, repair, and functioning of diverse tissues [13]. Our lab has used collagen-GAG scaffolds to study cellular activity in osteoblasts and MSC osteogenesis extensively [26,27,28,29]. Experiments have investigated the role of GAG compositions [26], concentration [27,30], and physical characteristics such as stiffness and pore size upon cellular attachment, proliferation, and migration of cells [27,30,31,32,33]. Moreover, these compositions can be fabricated in three-dimensions (3D), acting as ECM analogues. These analogues can be used to study the effects of fluid flow stimulation, modelling in vivo mechanical loading of bone cells [31,34,35]. Regarding osteogenesis, much of our knowledge has been gained from 2D culture studies, whereas 3D culture is now known to be far superior, better representing the cellular experience in vivo [36,37]. Bone demonstrates a unique three-dimensional ECM in the body. The main inorganic bone ECM element, hydroxyapatite (HA), has recently proven itself to not only act as a structural ECM component that confers strength and rigidity to bone, but also as a bioactive material [38]. Interestingly, HA has also been shown to modulate gene expression in osteocytes [39]. The addition of a hydroxyapatite mineral phase to form collagen-HA scaffolds has greatly increased our understanding of osteogenesis [11,40,41,42]. HA is both osteoinductive and osteoconductive, and advancing research has permitted the development of tissue engineered bone repairing constructs [40,41,43], which have proven themselves in vivo [44]. Given that sclerostin is understood to be produced by the mature osteocyte while embedded in its mineralised matrix, we postulate that a mineralised matrix analogue could also be applied as an ECM substitute, fostering the interactions we understand to be important for correct osteocyte signalling and coordination of bone adaptation.

The objective of this study was therefore to investigate the role of substrate composition and dimensionality in directing osteocyte *Sost* gene expression in the well-established cell line, the MLO-Y4. The hypothesis of this study is that these cells would behave more like a native osteocyte in a more physiologically relevant environment to mineralised bone. We postulate that culturing MLO-Y4 cells in ECMs mimetic to native bone ECM containing GAGs and HA, in both 2D and 3D culture will overcome some of the limitations of this cell line when compared to traditional collagen-coated plastic culture conditions.

## 2. Materials and Methods

### 2.1. Cell Culture

MLO-Y4 cells were used in this study; this cell line is a murine derived model of an osteocyte (gift from Dr. Lynda Bonewald, University of Missouri-Kansas City, MO, USA) [14]. MLO-Y4 cells were expanded on rat tail collagen (BD Biosciences, Bedford, MA, USA) coated cell culture plastic with α-Modified Eagle’s Media (α-MEM) supplemented with 5% calf serum (CS), 5% foetal bovine serum (FBS), 1% L-Glutamine, and 2% penicillin–streptomycin (P/S). The passage number for this study was 35–39.

### 2.2. Substrate Compositions

ECM analogues of varying compositions were produced using collagen slurries [45]. Established protocols for the incorporation of additional ECM components were followed [29,30,43]. For 3D scaffolds, this included well-characterised freeze-drying temperatures to ensure consistent pore sizes were produced [32,45,46,47]. The approaches are briefly described below.

#### 2.2.1. Collagen Only Slurry

A collagen slurry suspension was produced by mixing micro-fibrillar Collagen Type I (0.5% *w*/*v*) with 0.05 m glacial acetic acid (Fisher Scientific, Loughborough, UK). The suspension was then added to a mixing vessel (Lauda WKL230; Lauda Brinkman LP, Delran, NJ, USA), cooled to 4 °C to prevent protein denaturation, and blended at 15,000 rpm using an overhead blender (Ultra-Turrax T18; IKA Works Inc., Wilmington, NC, USA) until a homogeneous suspension was formed. The suspension was then degassed under vacuum and maintained at 4 °C [45].

#### 2.2.2. Collagen-Glycosaminoglycan Slurry

A collagen-glycosaminoglycan (GAG) slurry was produced by mixing micro-fibrillar Collagen Type I (0.5% *w*/*v*; Collagen Matrix, Allendale, NJ, USA) with chondroitin-6-sulphate sodium salt, isolated from shark cartilage (0.05% *w*/*v*; Sigma-Aldrich, St. Louis, MI, USA) in 0.05 m glacial acetic acid (Fisher Scientific, Loughborough, UK) [33,48,49]. The suspension was added to a mixing vessel (Lauda WKL230; Lauda Brinkman LP, Delran, NJ, USA), cooled to 4 °C to prevent protein denaturation, and blended at 15,000 rpm using an overhead blender (Ultra-Turrax T18; IKA Works Inc., Wilmington, NC, USA) until a homogeneous suspension was formed before degassing under vacuum and maintained at 4 °C.

#### 2.2.3. Collagen-Hydroxyapatite Slurry

The collagen-hydroxyapatite slurry was based on that used to create a novel scaffold developed within our group [50]. This scaffold has shown itself to demonstrate potent osteoinductive and osteoconductive capabilities in both in vitro and in vivo studies [43,50]. It has been realised as an adjunct to bone repair in a range of species through in vivo studies [44,48,49,51] and notably, most recently including humans. This scaffold slurry consists of type I microfibrillar bovine collagen (Collagen Matrix, Allendale, NJ, USA) and 0.5 M glacial acetic acid, blended together at 15,000 rpm using an overhead blender (Ultra Turrax T18, IKA Works Inc., Wilmington, NC, USA). Blending was carried out in a reaction vessel, which was maintained at 4 °C using a circulation cooling system (WKL 230, Lauda Brinkman LP, Delran, NJ, USA) for a total of five and half hours. HA powder (Capital R reaction powder, Plasma Biotal, Tideswell, UK) was suspended in 0.5 M acetic acid solution and added in aliquots every hour to the collagen suspension during blending. The resulting collagen slurry contained 0.5% (*w*/*v*) collagen and a HA concentration of 1% (*w*/*v*). The slurry was degassed in a vacuum and maintained at 4 °C [43].

#### 2.2.4. Collagen-Glycosaminoglycan-Hydroxyapatite Slurry

A collagen-glycosaminoglycan (GAG) hydroxyapatite (HA) slurry was produced by mixing micro-fibrillar Collagen Type I (0.5% *w*/*v*; Collagen Matrix, Allendale, NJ, USA) with chondroitin-6-sulphate sodium salt, isolated from shark cartilage (0.05% *w*/*v*; Sigma-Aldrich, St. Louis, MI, USA), and HA powder (1% *w*/*v*; Capital R reaction powder, Plasma Biotal, Tideswell, UK) in 0.05 m glacial acetic acid (Fisher Scientific, Loughborough, UK). The suspension was added to a mixing vessel (Lauda WKL230; Lauda Brinkman LP, Delran, NJ, USA), cooled to 4 °C to prevent protein denaturation, and blended at 15,000 rpm using an overhead blender (Ultra-Turrax T18; IKA Works Inc., Wilmington, NC, USA) for a total of five and half hours until a homogeneous suspension was formed before degassing under vacuum and maintained at 4 °C.

### 2.3. Fabrication of 2D Film ECM Analogues

**Figure 1:**
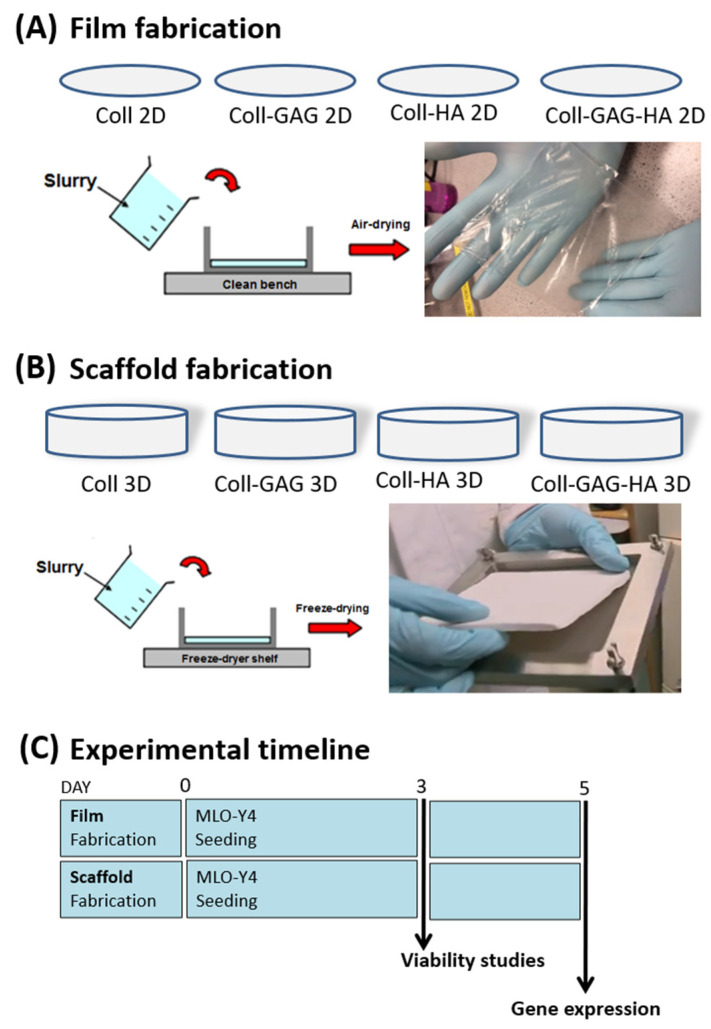
Experimental design. Slurries of four different compositions were produced: Collagen-1 (Coll), Collagen-1 + Chondroitin Sulphate (Coll-GAG), Collagen-1 + Hydroxyapatite (Coll-HA), Collagen-1 + Chondroitin Sulphate + Hydroxyapatite (Coll-GAG-HA). (**A**) 2D films were produced by allowing slurries to air dry upon a clean bench. (**B**) 3D scaffolds were fabricated by freeze drying slurry preparations. (**C**) Experimental timeline whereby all ECM analogues were seeded. After 3 days culture samples were analysed for viability assays. After 5 days seeded ECM analogues were analysed for differential gene expression between each composition groups, and between 2D and 3D culture groups.

In order to allow a comparative analysis between cells cultured on 2D film ECM analogues versus 3D scaffold analogues. The collagen slurries of different compositions mentioned above, were first fabricated into 2D films. Briefly, 62.5 mL of each respective slurry was added to a 125 × 125 mm stainless steel bracket that had been clamped to a PFTE plate, which acted as a mould. The slurry underwent dehydration over 48 h at room temperature in a fume hood (Figure 1A). Once dry, films were hydrated in PBS and punched into discs of 34 mm diameter using a stainless-steel punch (Gedore, Remscheid, Germany). Discs were allowed to dry on a PTFE plate in a fume hood before placement in foil sleeves for dehydrothermal crosslinking at 105 °C for 24 h in a vacuum oven at 50 mTorr (VacuCell, MMM, Planegg, Germany) (Figure 1A).

### 2.4. Fabrication of 3D Scaffold ECM Analogues

Three-dimensional culture has fast been realised as a superior strategy for the study of cells in vitro. The added dimension allows cellular polarisation and formation of apical and basal profiles with which to interact with matrix and adjacent cells. This has been realised in tissue engineering strategies investigating many different tissues such as those in bone, blood vessels, and the eye [11,13,19,36,37,47,52,53,54,55,56,57]. To create 3D scaffolds for this study, respective slurries were freeze-dried (VirTis Co., Gardiner, NY, USA) to a final freezing temperature of −10 °C [45]. After lyophilization, scaffold sheets were dehydrothermally crosslinked at 105 °C for 24 h in a vacuum oven at 50 mTorr (VacuCell, MMM, Planegg, Germany). Individual scaffold discs (diameter 12.7 mm; depth 3–4 mm) were punched out of the sheets. Scaffolds were stored in phosphate buffered saline (PBS; Sigma-Aldrich) (Figure 1B).

### 2.5. Cellular Seeding of MLO-Y4 Osteocytes upon 2D and 3D ECM Analogues

To seed materials, MLO-Y4 cells were detached from flasks with Trypsin-EDTA (Sigma-Aldrich) and resuspended in culture media (described above). Films and scaffolds were seeded with a total of 150,000 cells. In six-well plates, 100 µL of cell suspension was added dropwise onto the top surface of films or scaffolds, and the plates were placed into the incubator for 15 min to allow for cell attachment. Whilst scaffolds required no stabilisation during seeding in the wells, films were held in position using polycarbonate, CellCrown™ inserts (Scaffdex, Tempere, Finland) for 24 h after seeding. After this incubation period, 5 mL of culture media was added to each well. Seeded constructs were cultured under standard conditions (37 °C, 5% carbon dioxide). For compositional studies, constructs were carefully washed with phosphate buffered saline (PBS) 120 h following seeding and underwent RNA extraction (Figure 1C).

### 2.6. RNA Extraction and Gene Expression Analysis

Samples were blended in 1 mL Qiazol lysis reagent (QIAGEN, Frederick, MD, USA) using a homogeniser (Omni International, Kennesaw, GA, USA). Then, 200 μL chloroform (BDH, Poole, UK) was added to the lysate to facilitate phase separation. The Aqueous phase was then transferred to labelled RNeasy columns (RNeasy mini kit (QIAGEN) to extract total RNA as per manufacturer’s instructions. RNA was both qualified and quantified using Nanodrop 2000 (Thermo Scientific, Waltham, MA,, USA) that recorded light absorbance at 260 nm. QuantiTect RT Kit (Qiagen, Frederick, MD, USA) was used for reverse transcription to generate a cDNA library. This was followed by Real-time PCR (Eppendorf Mastercycler Realplex 4 System, Eppendorf, Hamburg, Germany) and QuantiTect SYBR Green PCR Kit (Qiagen, Frederick, MD, USA) using QuantiTect primers for Sost and with 18S used as a housekeeping gene (for primer sequences—see Supplementary Table S1).

### 2.7. Sost Expression in Murine Long Bones

Primary murine Sost expressing osteocytes were used as a control group. Three skeletally mature adult male mice were used. Animals had no prior intervention regarding exercise or weight bearing and were otherwise unrestricted in their housing. Osteocytes comprise the vast amount of bone cells with osteoprogenitors residing primarily upon the periosteal surface, whilst mesenchymal and haematopoietic stem cell niches reside primarily in the marrow. Cartilage and tendon connective tissue are localised towards the articulating surface. In order to maximise the RNA yield from osteocytes, RNA was isolated from the mice femoral shafts. Three adult mice were euthanised and the femora were quickly dissected and withdrawn. Soft tissue was removed from the bone, periosteum was stripped, and diaphysis isolated. Marrow was washed from the central cavity using warmed PBS before the bone was snap frozen in liquid nitrogen. Bone samples were subsequently blended in 1 mL Qiazol lysis reagent (Qiagen, Frederick, MD, USA) using a homogeniser (Omni International, Kennesaw, GA, USA). The lysate then underwent RNA extraction for use in RT-PCR analysis as described above.

### 2.8. Effect of Fluid Flow upon Sost Expression in MLO-Y4 Cells Cultured in Collagen-HA 3D Environments

MLO-Y4 cells seeded on collagen-HA scaffolds were pre-cultured for 72 h prior to random allocation to either a static or flow group. Static group constructs underwent a fresh media change and were cultured under standard conditions for a further 48 h, after which they underwent RT-PCR analysis. The flow group constructs were placed into customised individual flow perfusion bioreactors. Each reactor consisted of programmable syringe pumps, scaffold chambers, and media reservoir (containing 50 mL of growth media) (Figure 6B) [58]. The flow profile comprised of 1 h steady flow at 1 mL/min followed by 7 h low flow of steady flow at 0.05 m/min over 48 h [59]. After the 48 h culture period, the scaffolds were removed for RT-PCR analysis.

### 2.9. Statistical Analysis

Statistical analyses were performed using GraphPad Prism (Version 5.0) (GraphPad Software, San Diego, CA, USA) software. All data are expressed as means ± standard error with overlying data points. One-way analysis of variance (ANOVA) was used with a Bonferroni test to compare between groups. A probability value of 95% (*p* < 0.05) was used to determine significance.

## 3. Results

### 3.1. ECM Composition Influences Cellular Metabolic Activity

To determine the effect of substrate composition upon osteocyte viability and metabolic activity, MLO-Y4 cells were seeded on collagen-based 2D films consisting of: (i) collagen, (ii) collagen-GAG, (iii) collagen-HA, and (iv) collagen-GAG-HA. These will be referred to a Coll, Coll-GAG, Coll-HA, and Coll-GAG-HA, respectively. The main finding was that while cellular DNA did not change with the addition of ECM components, there was a significant cumulative reduction in cellular metabolic rate.

**Figure 2:**
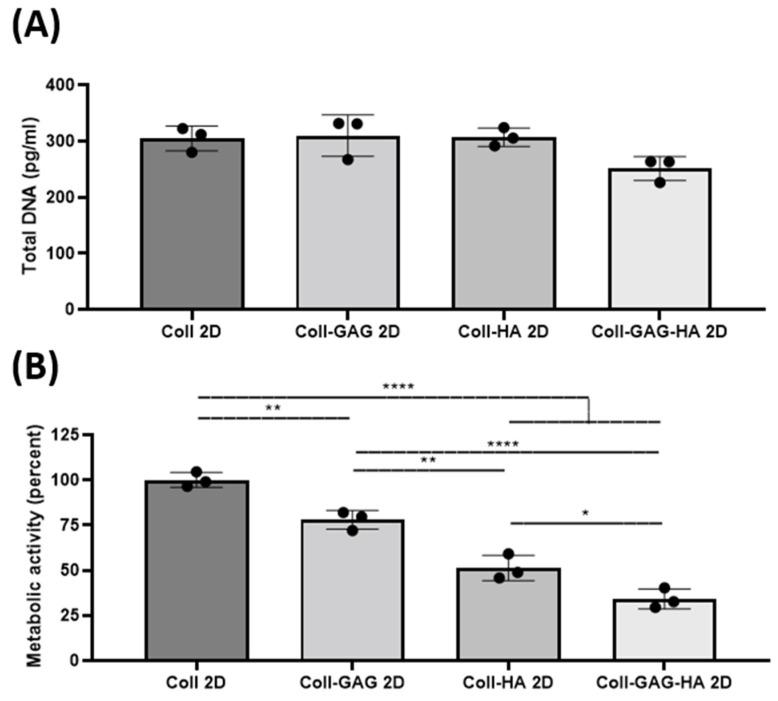
Changes in biological activity of MLO-Y4 cells cultured on different 2D film substrate compositions: Collagen-1 (Coll 2D), Collagen-1 + Chondroitin Sulphate (Coll-GAG 2D), Collagen-1 + Hydroxyapatite (Coll-HA 2D), Collagen-1 + Chondroitin Sulphate + Hydroxyapatite (Coll-GAG-HA 2D). (**A**) Proliferation as measured by DNA content. (**B**) Metabolic Activity as measured by MTS assay. (*n* = 3; * *p* < 0.05; ** *p* < 0.01, **** *p* < 0.0001).

The average total DNA in the Coll control group was 305 pg. The Coll-GAG composition 2D film was found to have the highest total DNA content at 310 pg. In comparison, the Coll-GAG-HA composition film was found to have the lowest average total DNA content compared to the other groups at 251 pg. However, no statistical difference was found between the groups (Figure 2A), indicating that 2D substrate composition does not impact on cell number/viability. Regarding cellular metabolism, all groups were normalised to Coll 2D control. The stepwise addition of GAG and HA components were found to reduce the metabolic activity of cultured MLO-Y4 cells. The Coll-GAG 2D metabolic activity was found to be reduced to 77.9% ± 5.2% (**, *p* = 0.0054), as was the Coll-HA 2D group at 51.3% ± 7% (****, *p* < 0.0001). The Coll-GAG-HA group had the lowest overall at 34.1% ± 5.5% (****, *p* < 0.0001) (Figure 2B).

Moreover, morphological analysis was performed on MLO-Y4 cells cultured on each film after 72 h. No difference was identified in average # of dendrites/cell, dendrite length, or average nuclear to cytoplasmic ratio across all film compositions (Figures S1 and S2).

Taken together, these data suggest that varying compositions did not alter cell viability or proliferation as DNA remained similar across groups and did not alter cell morphology, although ECM components significantly affected cellular metabolic rates.

### 3.2. ECM Substrate Composition Directs Sost Expression in MLO-Y4 Cells

**Figure 3:**
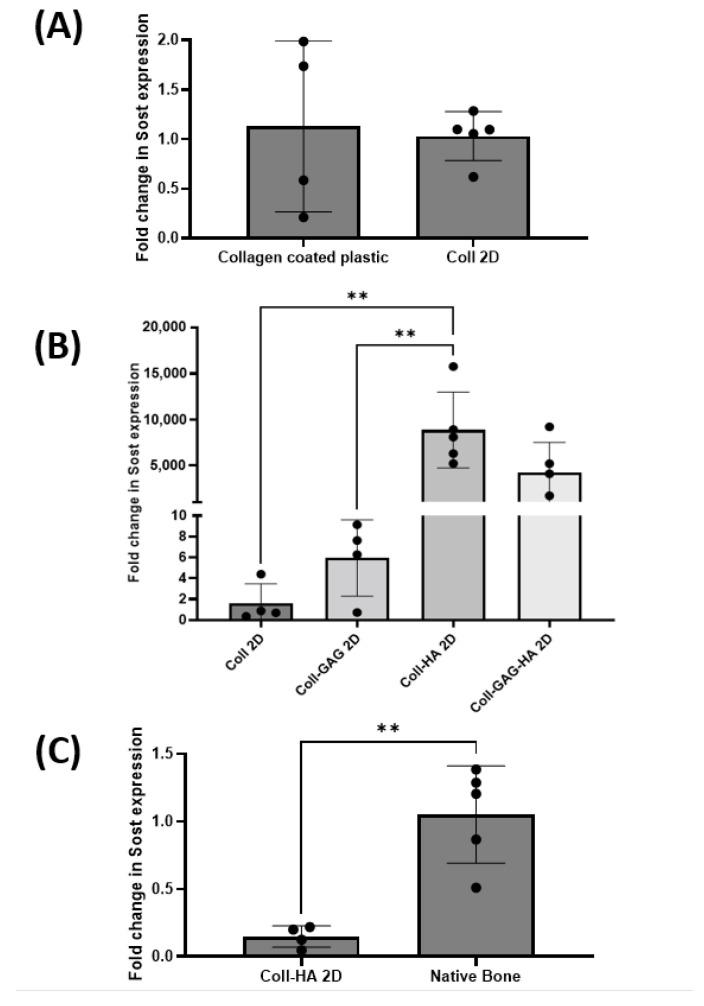
(**A**) MLO-Y4 *Sost* expression is comparable between 2D collagen coated cultures and collagen film cultures. (**B**) Films containing HA demonstrate highest levels of MLO-Y4 *Sost* expression. (**C**) MLO-Y4 *Sost* expression in Coll-HA films are significantly lower than those found in native bone. (*n* = 4–5; ** *p* < 0.01)**.**

To establish the effect of substrate composition upon osteocyte *Sost* gene expression, MLO-Y4 cells were seeded on Coll, Coll-GAG, Coll-HA, and Coll-GAG-HA 2D films as described above. Utilising *Sost* expression in MLO-Y4 cells cultured on 2D collagen-coated plastic as a reference, it was found that *Sost* expression was comparable to that of cells cultured on 2D collagen films (Figure 3A), suggesting that the increased thickness of collagen within the film did not impact on osteocyte signalling. The addition of GAG to the film triggered a trend towards an increase in MLO-Y4 *Sost* expression when compared to the collagen only film, with a 5.9-fold increase; however, this was not statistically significant at *p* = 0.0178. The highest expression of all groups was found in MLO-Y4 cells cultured on the Coll-HA film, with an 8868-fold increase, which was found to be statistically significant when compared to the Coll-GAG and Coll control films (*p* < 0.01) (Figure 3B). This very large fold increase speaks largely to the negligible expression in MLO-Y4 cells cultured under standard conditions. Expression was also enhanced in the Coll-GAG-HA group, with a 4202-fold increase when compared to the Coll control; however, this was not significant.

Given this significant increase in *Sost* expression on the Coll-HA films, we next compared expression to that in murine long bone. Despite this improved expression of *Sost* in the MLO-Y4 cell line, it is still significantly lower than physiological levels (*p* < 0.01) (Figure 3C). These data taken together indicate that substrate composition, specifically the presence of HA, can direct the expression of *Sost* in MLO-Y4 cells, but not at a level to that found in vivo.

### 3.3. ECM Dimensionality Influences Cellular Metabolic Activity

**Figure 4:**
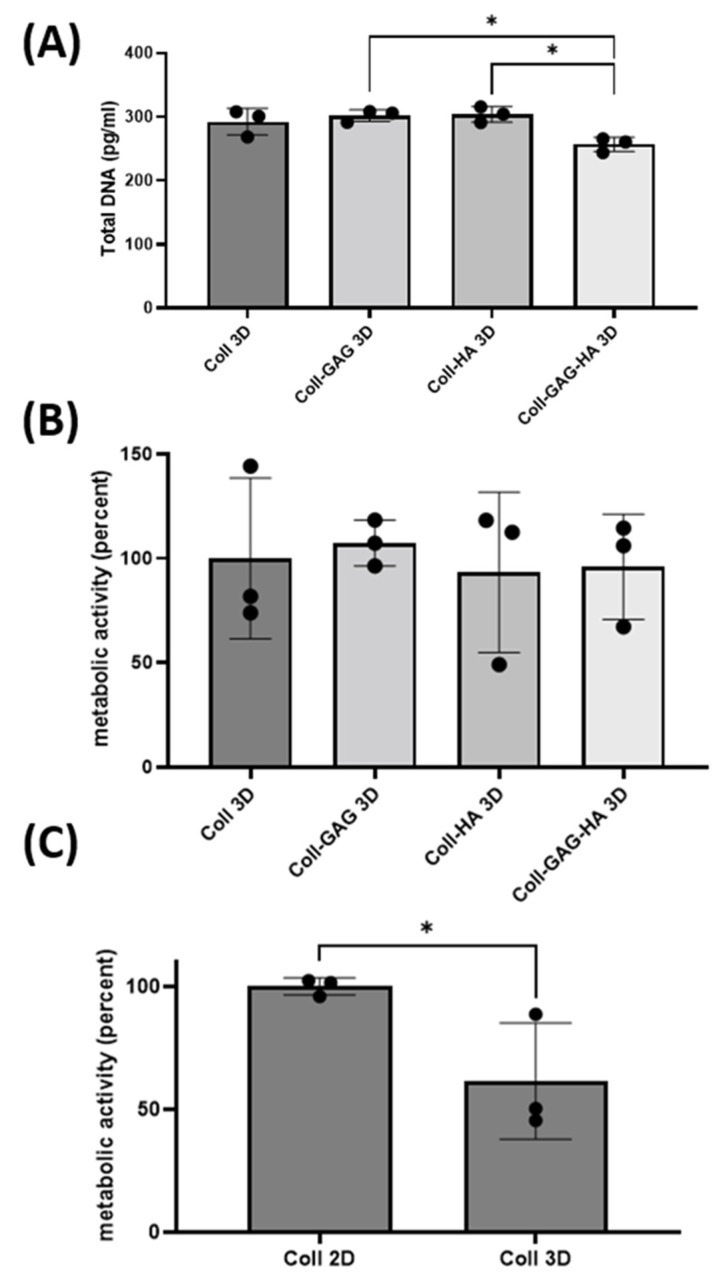
Changes in biological activity of MLO-Y4 cells cultured on different 3D scaffold substrate compositions: Collagen-1 (Coll 3D), Collagen-1 + Chondroitin Sulphate (Coll-GAG 3D), Collagen-1 + Hydroxyapatite (Coll-HA 3D), Collagen-1 + Chondroitin Sulphate + Hydroxyapatite (Coll-GAG-HA 3D). (**A**) Proliferation as measured by DNA content. (**B**) Metabolic activity as measured by MTS assay. (**C**) Metabolic activity in cells cultured on Coll 2D versus Coll 3D. (*n* = 3; * *p* < 0.05).

To investigate the impact of dimensionality, the ECM compositions investigated earlier were fabricated into 3D porous scaffolds and were utilised as a platform for MLO-Y4 culture. The 3D scaffold with the highest DNA content was the Coll-HA 3D composition at 303 pg whist the lowest value was found in the Coll-GAG-HA 3D group at 256 pg, which is consistent with trends identified on 2D films. While the Coll-GAG 3D and Coll-HA 3D scaffolds did not demonstrate a significant difference in DNA when compared to Coll 3D, the DNA in these groups was significantly higher than that measured in Coll-GAG-HA 3D (*p* < 0.05) (Figure 4A).

Regarding metabolic activity, no statistical difference was identified between the 3D ECM groups (Figure 4C), which is inconsistent with that identified in 2D, where the addition of ECM components reduced the activity of MLO-Y4 cells (Figure 4B). Interestingly, when comparing metabolic activity in 2D vs. 3D using the Collagen alone substrate, there is a significant decrease in cellular metabolic activity when cultured in 3D (*p* < 0.05) (Figure 4C), indicating that dimensionality influences the metabolism of MLO-Y4 cells.

### 3.4. 3D Scaffold Culture Increases Osteocyte Sost Expression When Compared to 2D Film Counterparts

To investigate the effect of substrate dimensionality upon *Sost* gene expression, MLO-Y4 cells were seeded on collagen-based 3D scaffolds supplemented with different ECM components as described above.

**Figure 5:**
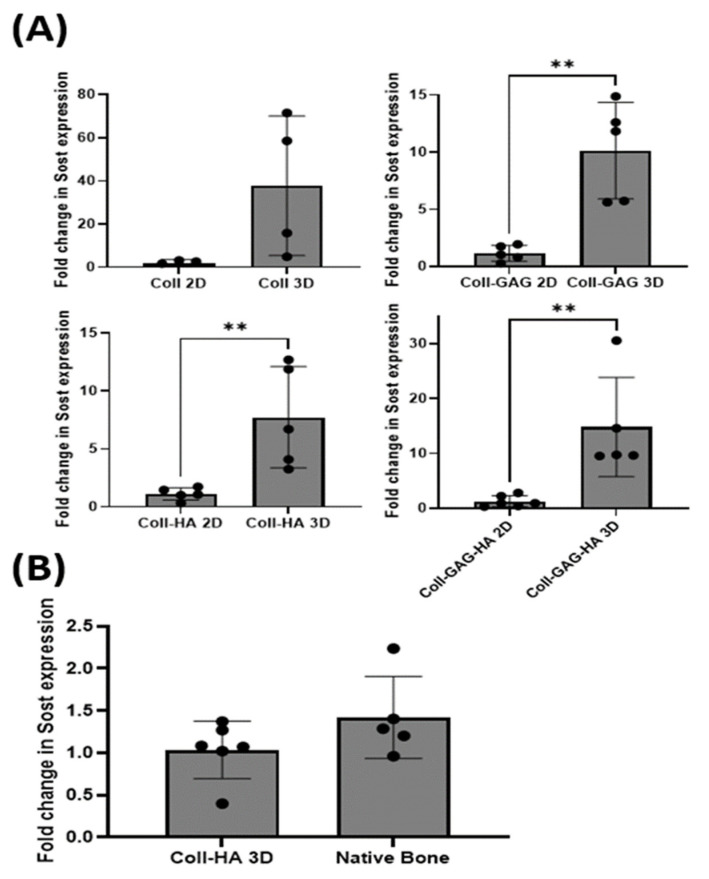
(**A**) *MLO-Y4 Sost* expression was higher in cells cultured on 3D ECM scaffolds relative to those on 2D thin films for all group composition studied. (**B**) No statistically significant difference existed between the *Sost* expression of cells cultured on Coll-HA 3D scaffolds compared to native bone. (*n* = 4–6; ** *p* < 0.01)**.**

Osteocytes cultured on Coll 3D scaffolds demonstrated an increased albeit insignificant *Sost* expression when compared to its Coll 2D film counterpart, with a 37.6-fold increase (*p* = 0.067). Cells cultured on Coll-GAG 3D scaffolds demonstrated a significant increase in *Sost* expression compared to the Coll-GAG 2D, 10.1-fold increase (*p* < 0.01). Interestingly, MLO-Y4 cells cultured on Coll-HA 3D scaffolds also demonstrated a further significant increase in *Sost* expression compared to the Coll-HA 2D film, 7.7-fold increase (*p* < 0.01), which had previously demonstrated that best response in 2D. Finally, osteocytes cultured on Coll-GAG-HA 3D scaffolds demonstrated a significant increase in *Sost* expression compared to the Coll-GAG-HA 2D film, 14.8-fold increase (*p* < 0.01) (Figure 5A). These data taken together demonstrate that 3D culture can significantly enhance the expression of *Sost* in the MLO-Y4 osteocyte cell line when compared to cells cultured upon 2D ECM analogues.

### 3.5. MLO-Y4 Osteocyte-like Cells Express Physiological Levels of Sost in Coll-HA 3D Environments

The level of *Sost* expressed by MLO-Y4 osteocyte cells cultured on a Coll-HA 3D scaffolds was compared to that expressed in murine long bone. Interestingly, no statistical difference was found between the two groups investigated (*p* = 0.15) (Figure 5B). These data demonstrate that MLO-Y4 cells cultured on collagen-HA scaffolds may act as an in vitro model system capable of expressing Sost in comparable levels to primary cells in vivo.

### 3.6. Sost Expression in Coll-HA 3D Osteocyte Seeded Scaffolds Decreases in Response to Fluid Flow

**Figure 6:**
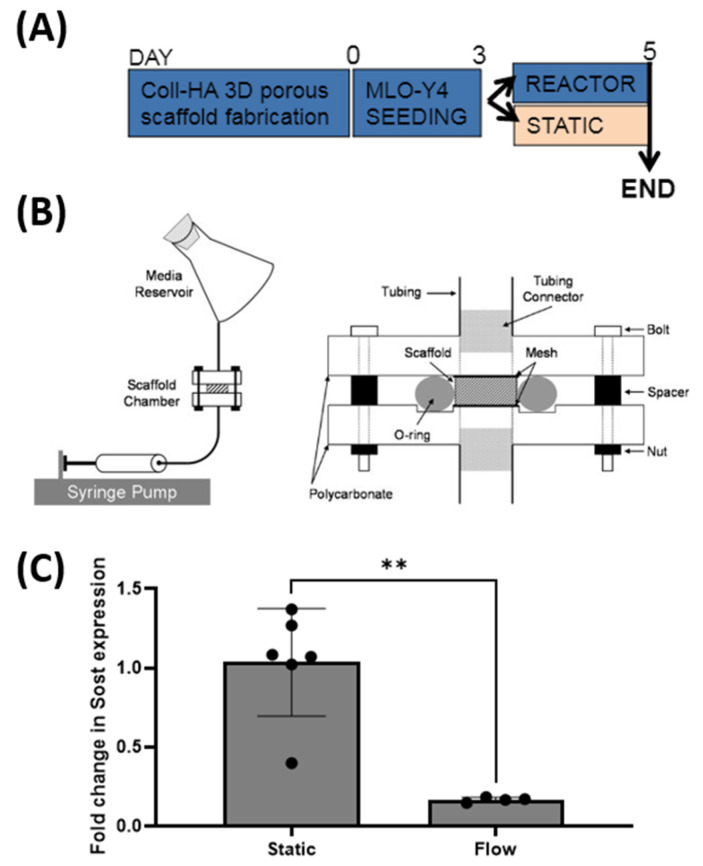
(**A**) Timeline for flow stimulation experiment. (**B**) Schematic of flow perfusion reactor. (**C**) *Sost* expression by MLO-Y4 cells cultured collagen-HA scaffold subjected to flow stimulation versus statically cultured. *Sost* expression decreased significantly upon flow stimulation (**, *p* < 0.01).

We next sought to investigate whether *Sost* expression is functionally regulated by mechanical stimulation as is seen in vivo. A perfusion bioreactor was utilised to investigate the effects of fluid flow upon MLO-Y4 cells seeded within the Coll-HA 3D scaffold (Figure 6A). Flow-stimulated constructs demonstrated a significant drop in *Sost* expression when compared to statically cultured controls at 0.16-fold expression relative to the control (equating to an 83% decrease), (*p* < 0.01) (Figure 6C). Therefore, these data suggest that *Sost* expression by MLO-Y4 cells cultured on Coll-HA 3D scaffolds is appropriately regulated by mechanical stimulation akin to that of primary osteocytes in vivo.

## 4. Discussion

Bone formation is a complex process regulated in part by the osteocyte specific protein sclerostin. A main cell line developed for studying osteocytes, the MLO-Y4 cell, does not produce this protein in physiological amounts under standard culture methods. The overall objective of this study was therefore, to investigate if substrate composition and/or dimensionality could influence the expression of *Sost* in MLO-Y4 cells. A significant outcome of this study is that MLO-Y4 cells were found to express *Sost* when cultured on a HA containing 2D film analogues. Three-dimensional culture upon HA containing scaffolds further increased *Sost* expression. Importantly, *Sost* expression in MLO-Y4 cells cultured on a Coll-HA 3D scaffold was found to be comparable to physiological levels. Moreover, MLO-Y4 cells cultured in these novel conditions responded accordingly to fluid flow stimulation with a decrease in *Sost* expression, indicating that *Sost* expression is regulated in these novel culture conditions in a similar manner to that seen in vivo. This study therefore presents a novel culture system for the MLO-Y4 osteocyte cell line ensuring the expression of an important osteocyte specific gene, *Sost*, overcoming the main limitation of this model. Moreover, this study furthers our understanding of the need for appropriate biochemical and biophysical cues (i.e., substrate composition and dimensionality) for in vitro models of cell physiology. This has interesting further implications on preceding 2D MLO-Y4 studies, which represent the bulk of osteocyte research, in addition to future studies involving co-cultures with cells from both the mesenchymal and haematopoietic lineages. Such multicellular systems could be used to recapitulate physiological and pathological processes, in addition to acting as a possible stepping stone to in vivo studies, therefore engaging the replacement, reduction, and refinement principles of Russell and Burch [60].

This study demonstrated that substrate composition influences the expression of sclerostin. Traditional culture of MLO-Y4 cells is carried out on collagen-coated plastic to maintain a dendritic phenotype [14]. The addition of GAG, an ECM component, resulted in an increase in *Sost* expression relative to a collagen only control. GAG comprises an important part of the osteocyte glycocalyx and is required for correct mechanotransduction [61]. The addition of hydroxyapatite to the collagen-GAG construct resulted in further increased expression of *Sost*. This level of expression was not as high, however, when compared to the robust expression observed in the collagen-hydroxyapatite film alone. The HA concentration is the same in both collagen-GAG-HA and collagen-HA films, suggesting that HA is driving an enhanced *Sost* expression, which is being somewhat reduced by the presence of GAG, possibly by competitive binding with HA. This observation may also be attributed to the enhanced stiffness of the HA containing films due to the presence of the ceramic [62]; however, the response cannot be simply driven by a change in stiffness, as tissue culture plastic is in the GPa range [63] and, yet, fails to elicit a *Sost* response, indicating a biochemical component underlying the interaction.

Whilst HA has been established as an osteoconductive material, its osteoinductive potential has also been investigated [11,40,64]. This specifically refers to its ability to direct an undifferentiated cell down an osteogenic lineage rather than supporting the function of an already differentiated osteoblast. Lin et al., in 2008, using C3H101/2 cells, suggested that media conditioned by cells exposed to HA contained a secreted factor that could prime osteogenesis in cells. This cell–HA media was found to be 60% more effective than BMP supplemented media and 10x more effective than HA conditioned media alone [65]. Whilst Lin determined that this response was not simply due to leached Ca and Pi ions, other studies have attributed the osteoinductive effect to them [66]. Lin’s work adds to increasing research suggesting HA can act as a focal point for growth factors or potential extracellular vesicles (EVs) [67], forming an osteoinductive niche [65]. We can infer that as a stem cell and osteoblast supporting material, HA would also serve to facilitate and support the activity of further differentiated osteocytes. This hypothesis is also supported by the observation that the MLO-A5 post-osteoblast cell line can express the *Sost* gene in the presence of hydroxyapatite mineral in 3D culture conditions after only 24 h, which would suggest the environment does indeed play an important role in its regulation [39]. The expression of *Sost* by the IDG-SW3 cell in 2D culture is after approximately 10 days culture. This prolonged culture period may reflect the time required for the maturing osteocyte to lay a rudimentary mineralised matrix with which to interact. The significant role of osteocytes in remodelling and mineral homeostasis has been well described [9,10]. Moreover, these data add to the growing evidence of HA as a bioactive molecule that has been shown to modulate expression of osteocyte proteins, such as Frizzled, Dickkopf, and Wnt, which are master regulators of the planar cell polarity and canonical pathways in osteocytes [38]. Therefore, it is clear that the addition of endogenous bone macromolecules in the form of a more ‘bone-like’ mimetic ECM has a significant effect upon osteocyte gene expression, and that such culture conditions, in the form of Coll-HA, can improve the power of the MLO-Y4 osteocyte model in emulating the primary osteocyte.

Interestingly, whilst the sequential addition of substrate components resulted in significant increases in *Sost* expression, there was a corresponding significant decrease in the cellular metabolic rate. This observation may suggest that *Sost* expression is inherently linked to cellular metabolism in this culture model. Given that MLO-Y4 cells are highly active and dividing cells, we can infer that proliferation would represent the majority of the metabolic burden occurring within a cell [68]. Cellular division is one such way the MLO-Y4 differs from terminally differentiated primary osteocytes that do not divide. Together, this may suggest that cells cultured upon bone-like ECMs were encouraged to exit the highly metabolically active proliferation cycle, and instead temporarily slow to facilitate a more differentiated osteocyte genotype. This observation may also be traced back to the development of the cell line itself. It is described that cells cultured upon plastic lose their dendritic phenotype, whereas cells cultured on collagen-coated plastic maintain their dendrites, but interestingly also have a markedly reduced rate of proliferation [69]. The theory is further supported by the observations of our dimensionality studies. Here, it was demonstrated that cells cultured on collagen 3D substrates were found to have statistically significant lower metabolic rates when considered against their 2D counterparts. This drop in global metabolic activity when switching from a 2D to a 3D culture has been described previously in the literature in other cell types such as nephrons [53] and cancer cells [47]. Given the only difference between the identical substrate compositions is their dimensionality, we are reminded that native osteocytes reside in a 3D bone environment and the Coll 3D ECM would more closely model this native environment. A reduced metabolic rate was exhibited by, and comparable between, all cells cultured on 3D substrates. Moreover, these rates were found to emulate the metabolic rate of cells cultured on 2D HA containing films. Taken together, this suggests that dimensionality, and HA, have comparable but not cumulative effects upon osteocyte metabolism.

The impact of dimensionality upon osteocyte culture becomes more intriguing when we consider its role in directing cell *Sost* gene expression. Interestingly, there was an increase in gene expression when compared between 3D and 2D for all compositions. Indeed, 3D culture may well represent a critical element of osteogenesis. Notably, in 2D osteogenic cultures, cells are known to form condensations within which osteogenesis is maximised. Kim and Adachi have previously utilised rotary culture systems to generate osteogenic spheroids using human mesenchymal stem cells (MSCs) [70]. Importantly, they have also utilised this culture system with osteoblastic MC3T3 cells that underwent osteogenic differentiation, importantly, without exogenous osteogenic supplements [71]. This highlights the role of mechanical cues in osteogenesis. More recently they have also demonstrated the role of hypoxia in this culture system for accelerating osteogenesis and *Sost* expression [72]. In this study, the greatest variability in expression occurred, understandably, in the collagen alone culture groups, as we expect only trace expression in these cells. Importantly, whilst there was no consistent predictable fold change in Sost expression, the increase was observed in all groups. Notably, this difference was quite robust, such that the remaining three groups were statistically significant. Reflecting upon this variability of enhanced expression, we are reminded of the possible competitive ligand binding previously discussed above. Here, competition exists for GAG and HA to bind and interact with cell receptors such as integrins. This primary order competition between ligand/receptors may be expanded to greater second order interactions, such as ligand multivalency; that is, the number of ligands organised within a nanoscale cluster, which can exert complex cellular effects [73,74,75]. Both GAG and HA would be expected to interact with cell receptors through clustering and statistical rebinding and their co-location may account for the larger fluctuations in Sost expression in GAG-HA ECMs compared to HA alone, whereby critical HA orientations and binding confirmations are less efficacious due to interpositioned GAG molecules. This explanation does not take into account the formation of contrasting focal adhesions and inter-receptor communication such as orientation, stoichiometry, and sub-cellular location of signalling molecules which we know are essential for normal osteocyte functions such as mechanosensors [9,39,61,76,77].

Our data suggest that the high expression of Sost in cells cultured on the Coll-HA 3D ECM analogue is comparable to that of bone cells in vivo. Our lab has previously shown that Coll-HA scaffolds can act as an excellent bone mimetic environment for the support of osteoblasts and stem cells in vitro [40,41,43]. The material stiffness acts as both a mechanical cue for osteogenic differentiation [78], whilst also overcoming contracting forces that allow the porous nature to remain patent and encourage the proliferation and migration of cells throughout [43,79]. This has been realised in in vivo environments as an adjunct to support and enhance bone repair [40,44,49,51]. These additional data demonstrate positive trophic effects for MLO-Y4 cells and encourage previously unobserved *Sost* expression, further validating the use of Coll-HA 3D as an in vitro model for investigating osteocyte physiology.

With osteocytes seeded upon 3D Coll-HA scaffolds expressing comparable levels of Sost to native cells, the next consideration was the effect of fluid flow upon this expression. This was achieved using a dynamic fluid flow bioreactor, previously validated in our lab for modelling in vivo mechanical loading stimulation [34,58]. Using the same dynamic flow regime, MLO-Y4 cells cultured on 3D Coll-HA scaffolds demonstrated a drop in *Sost* expression in response to fluid flow stimulation. The robust, statistically significant inhibition of *Sost* confirms that within 72 h culture time the osteocytes had formed the required cell–matrix connections with which to function in a physiological manner. Whilst the mechanisms of mechanosensing are incompletely understood, we can infer that the established well-described mechanisms of αvβ3 integrin binding of cellular processes and the primary cilium-mediated mechanosensing mechanism must be functional [76,80,81,82]. Taken together, these data support the Coll-HA 3D culture method as a model of efficiently encouraging the expression of the highly specific osteocyte genotype, comparable to that found in vivo, and observing normal physiological responses to mechanical stimulation through functional mechanosensors, and further validating the culture system as an in vitro model for investigating osteocyte physiology and mechanobiology.

A limitation to this study was the use of bone diaphysis lysate as the primary cell source. Whilst there are established protocols for the extraction of osteocytes, the requirements of this study were to obtain *Sost* expressing cells, which was achieved efficiently without the application of collagenases or prolonged hypoxia that could rapidly impact osteocyte gene expression. Given that no other bone cells are known to express *Sost*, and that osteocytes are by far the most abundant cell in bone diaphysis [83], we are confident that the extraction accurately represents expression in vivo. A further limitation is that the shear–stress magnitude generated by the fluid flow bioreactor is lower than that estimated to occur in vivo. It is possible, therefore, that there could be a more dramatic change in gene expression. In spite of this, we demonstrated a statistically significant drop in *Sost* with fluid flow, such as we would expect to observe in vivo.

## 5. Conclusions

This study has demonstrated the importance of substrate composition and dimensionality in directing cellular physiology; specifically, the *Sost* gene in the osteocyte-like MLO-Y4 cell line, which has traditionally been understood to have lost this ability. It is reasonable to infer that cells cultured on substrates with characteristics resembling the biophysical and biochemical properties of their in vivo environment would facilitate the optimised function of differentiated cell types. This ‘loss-of-function’ of differentiated cells in in vitro culture may be considered a type cellular ‘homesickness’, whereby the cells does not have the required biochemical and biophysical cues with which to demonstrate normal physiological behaviour. This indicates that cells can adopt increasingly complex genotypes under different culture conditions, and this can facilitate the expression of tissue-type specific genes and, consequently, have ramifications upon other in vitro studies involving differentiated cells and cellular homesickness, and this should therefore, in particular, be considered when planning any observational experiments with cell–matrix interactions. Additionally, this study has determined that the *Sost* gene is regulated by both composition and dimensionality of the ECM substrate, implying that activation of different cell–matrix interactions are capable of supporting and directing different functions. Lastly, we have established an in vitro model capable of expressing *Sost* at physiological levels that demonstrates normal behaviour in response to mechanical stimulation. This represents a useful tool for the investigation of osteocyte physiology in the future and could be adapted for future studies involving co-cultures with cells from both the mesenchymal and haematopoietic lineages. These multicellular systems could be used to further characterise environmental biophysical and biochemical cues, mediated by cell–cell, and cell–matrix interactions, under static and flow conditions. Together, such models could act as an intermediate investigatory step prior to in vivo studies, therefore realising the 3R principles of Russell and Burch [60].

## Data Availability

The data presented in this study are available on request from the corresponding author.

## Supplementary Materials

The following supporting information can be downloaded at: https://www.mdpi.com/article/10.3390/bioengineering9010035/s1, Table S1: Table related to primer sequences and concentrations employed in quantitative PCR analysis; Figure S1: High magnification or MLO-Y4 cells cultured of different substrates of (A) Collagen-1 (Coll 2D), (B) Collagen-1 + Chondroitin Sulphate (Coll-GAG 2D), (C) Collagen-1 + Hydroxyapatite (Coll-HA 2D), (D) Collagen-1 + Chondroitin Sulphate + Hydroxyapatite (Coll-GAG-HA 2D). (scale = 10um); Figure S2: Morphological data from cells cultured on 2D substrates for 72 h. (A) There were no significant differences in total number of dendrites per cells cultured upon different substrates. (B) There were no significant differences in the length of dendrites when cells were cultured upon different substrates. (C) There were also no significant differences in the nuclear to cytoplasmic to ratio of cells when cultured upon different substrates.
