# Supplementary material for: The Impact of the Extracellular Matrix Environment on Sost Expression by the MLO-Y4 Osteocyte Cell Line
Source: Bioengineering (Basel). 2022 Jan 13;9(1):35. doi: 10.3390/bioengineering9010035 (PMC8772728; doi:10.3390/bioengineering9010035)
Supplement: Supplementary file 1 [file bioengineering-09-00035-s001.zip › bioengineering-1519981-supplementary.pdf]

bioengineering

Supplementary

The Impact of the Extracellular Matrix Environment on *Sost* Expression by the MLO-Y4 Osteocyte Cell Line

Robert T. Brady [1,2,3,4], Fergal J. O'Brien [1,2,3], David A. Hoey [2,3,4,*]

1 Tissue Engineering Research Group, Department of Anatomy & Regenerative Medicine, Royal College of Surgeons in Ireland, Dublin, D02 YN77, Ireland. robertthomasbrady@gmail.com (R.T.B.); fjobrien@rcsi.ie (F.J.O'B)
2 Trinity Centre for Biomedical Engineering, School of Engineering, Trinity College Dublin, Dublin, D02 PN40, Ireland
3 Advanced Materials and BioEngineering Research Centre (AMBER), Trinity College Dublin & Royal College of Surgeons in Ireland, Dublin, D02 PN40, Ireland
4 Department of Mechanical, Manufacturing, and Biomedical Engineering, Trinity College Dublin, Dublin, D02 PN40, Ireland
* Correspondence: dahoey@tcd.ie

Table S1. Table related to primer sequences and concentrations employed in quantitative PCR analysis.

Gene symbol .	Tm (°C)	Primer concentration	Sequence	Amplicon Size
18s	65	400 nM	5′-GTAACCCGTTGAACCCCATT-3′	151bp
			3′-CCATCCAATCGGTAGTAGCG-5′	
Sost	60	400 nM	5′-CGTGCCTCATCTGCCTACTT-3′	185bp
			3′-ATAGGGATGGTGGGGAGGT-5′	

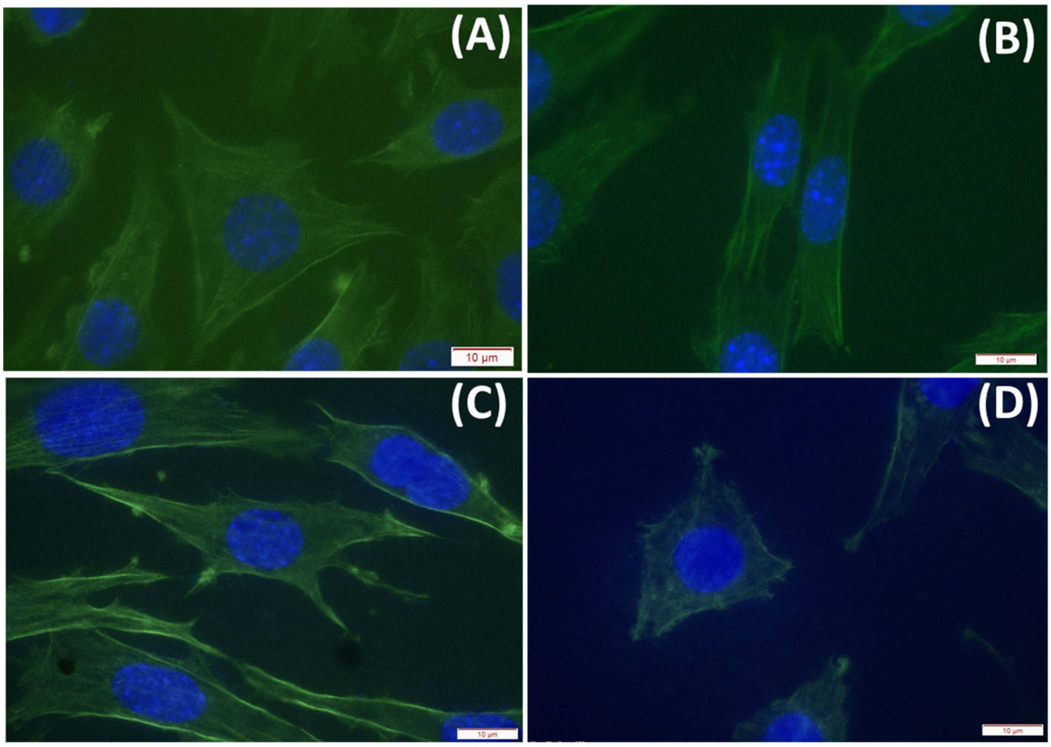

Figure S1. High magnification or MLO-Y4 cells cultured of different substrates of **(A)** Collagen-1 (Coll 2D), **(B)** Collagen-1 + Chondroitin Sulphate (Coll-GAG 2D), **(C)** Collagen-1 + Hydroxyapatite (Coll-HA 2D), **(D)** Collagen-1 + Chondroitin Sulphate + Hydroxyapatite (Coll-GAG-HA 2D). (scale = 10um).

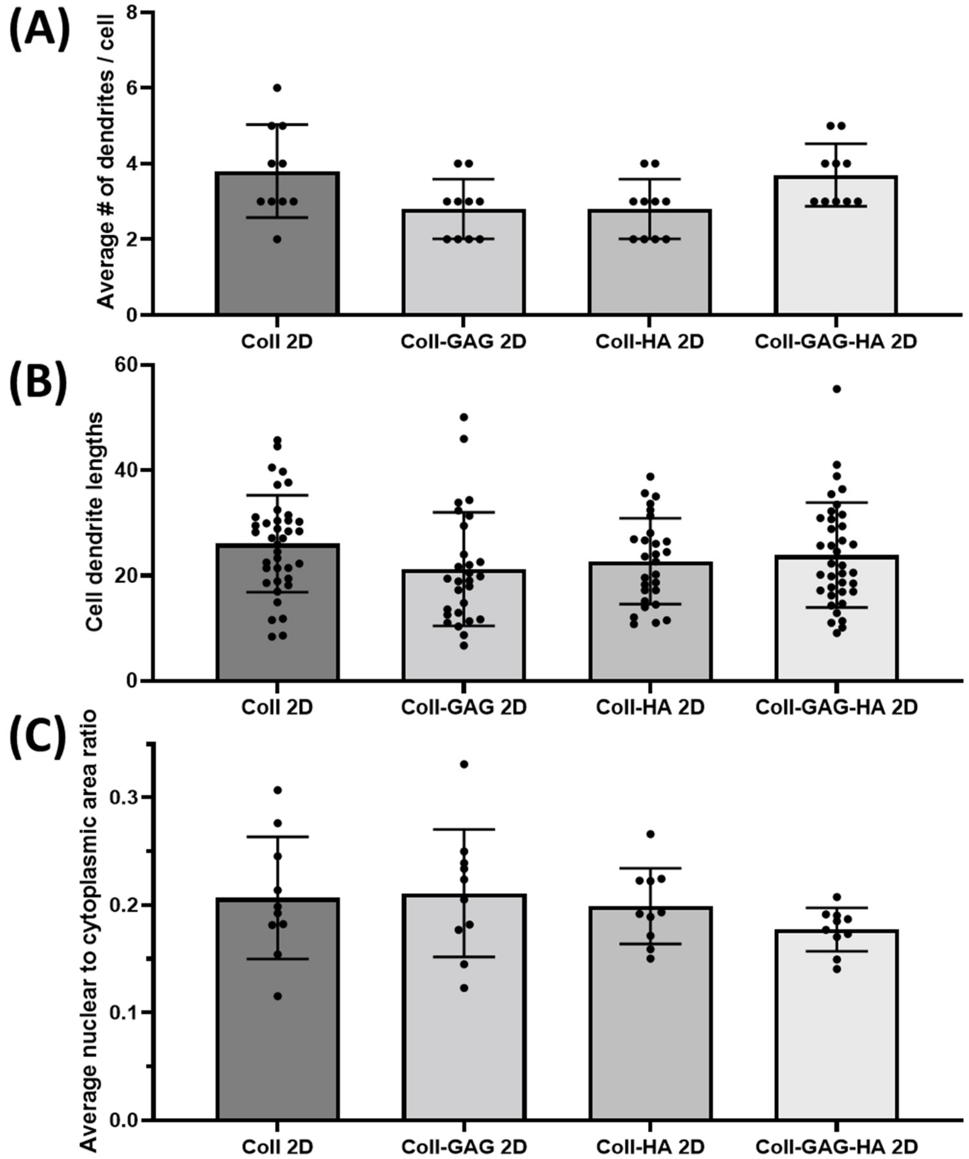

Figure S2. Morphological data from cells cultured on 2D substrates for 72 hours. **(A)** There were no significant differences in total number of dendrites per cells cultured upon different substrates. **(B)** There were no significant differences in the length of dendrites when cells were cultured upon different substrates. **(C)** There were also no significant differences in the nuclear to cytoplasmic to ratio of cells when cultured upon different substrates.
